# Ovarian transcriptomic study reveals the differential regulation of miRNAs and lncRNAs related to fecundity in different sheep

**DOI:** 10.1038/srep35299

**Published:** 2016-10-12

**Authors:** Xiangyang Miao, Qingmiao Luo, Huijing Zhao, Xiaoyu Qin

**Affiliations:** 1 Institute of Animal Sciences, Chinese Academy of Agricultural Sciences, Beijing, 100193, China

## Abstract

miRNAs and lncRNAs, which represent one of the most highly expressed classes of ncRNAs in development, are attracting increasing interest. A variety of regulators is considered to be implicated in sheep species with different fecundity. However, interactions between miRNAs and lncRNAs and changes in the expression of regulatory lncRNAs in sheep fecundity have not yet been reported. To characterize the important roles of miRNAs and lncRNAs and elucidate their regulating networks in sheep prolificacy, a genome-wide analysis of miRNAs and lncRNAs from Small Tail Han sheep of genotypes FecB^B^FecB^B^ (Han BB) and FecB^+^ FecB^+^ (Han++) and from Dorset sheep (Dorset) was performed. An integrated analysis of miRNAs and lncRNAs was performed to study the regulatory function of miRNAs and lncRNAs in fecundity, revealing significantly correlated patterns of expression. Dramatic changes of miRNAs and lncRNAs suggest their critical roles in sheep fecundity. In conclusion, this is the first study performing thorough investigations of regulatory relationships among lncRNAs, miRNA and mRNAs, which will provide a novel view of the regulatory mechanisms involved in sheep fecundity. These results may provide further insight into sheep fecundity and help us to improve sheep prolificacy.

Sheep (*Ovis aries*) are a highly diverse species raised for milk, skin, meat and fibre. Therefore, sheep prolificacy has been focused on for a long time. Small Tail Han sheep is a Chinese breed with hyperprolificacy but a slower growth rate^1^,^2^. Dorset sheep are widely bred in the US with a rapid growth rate but low prolificacy^3^,^4^,^5^,^6^. Importantly, ovulation rate and litter size are absolutely associated with sheep fecundity, and they have been shown to be affected by several genes, including bone morphogenetic protein receptor type 1B (*BMPR1B*), growth differentiation factor 9 (*GDF9*), X-linked maternally imprinted gene (*FecX2*) and beta-1,4-N-acetyl-galactosaminyltransferase 2 (*B4GALNT2*)^7^,^8^.

Epigenetic mechanisms include histone modification, DNA methylation and non-coding RNAs. Recently, the characterization of non-coding RNAs, including microRNAs (miRNAs) and long non-coding RNAs (lncRNAs), has become a fruitful area of research in animals and plants. In our previous work, we have found several novel miRNAs, such as miR-376d, miR-412-3p and miR-323c, that might play important roles in sheep development^9^. Moreover, another miRNA, oar-miR-665-5p, has been suggested to be differentially expressed in Han BB sheep compared with Dorset sheep and is negatively correlated with 3-hydroxy-3-methylglutaryl-CoA reductase (*HMGCR*), estrogen receptor 1 (*ESR1*), pyrroline-5-carboxylate reductase 1 (*PYCR1*) and glutamyl-prolyl-tRNA synthetase (*EPRS*), which are associated with the modulation of cellular cholesterol metabolism, proliferation and differentiation, oxidative stress response and inflammatory gene expression^10^,^11^,^12^,^13^. LncRNAs represent one of the most highly expressed ncRNAs in animals and regulate the expression of neighbouring coding genes. Considering the pivotal roles of lncRNAs in development and disease progression, we focused on their functions in sheep fecundity. Increasing evidence has indicated that the misregulation of lncRNAs is associated with various types of cancers^14^. Large numbers of lncRNAs have been detected using large-scale analyses of full-length cDNA sequences, and they exert critical roles in cell differentiation, development, immune responses and tumourigenesis^15^,^16^,^17^. Recent studies have suggested that in addition to targeting protein-coding RNAs, lncRNAs can also be directly targeted by miRNAs for cleavage^18^,^19^. It is valuable for us to investigate the critical role of lncRNAs in sheep fecundity. However, sheep-fecundity-associated miRNAs, lncRNAs and their regulatory networks remain unknown.

To investigate the potential role of lncRNAs in regulating sheep fecundity and to build miRNA, mRNA and lncRNA interaction networks in sheep, we performed RNA-seq to identify genome-wide differentially expressed genes (DEGs), miRNAs and lncRNAs in each comparison. Gene Ontology (GO) and Kyoto Encyclopedia of Genes and Genomes (KEGG) enrichment analyses of DEGs and target transcripts of miRNAs were conducted. We further constructed miRNA-lncRNA-mRNA interaction networks associated with sheep prolificacy to determine interactions among miRNAs, lncRNAs and mRNAs.

## Results

### Genome-wide identification of differentially expressed mRNAs, lncRNAs and miRNAs from sheep

Sequencing of all cDNA and small RNA libraries generated 156,412,756 raw paired-end reads with a length of 100 bases, resulting in a total of 16.7 gigabases (Table 1). From the expression profiles, differentially expressed mRNAs, lncRNAs and miRNAs were discriminated between two comparison groups (Table 2). Among these differentially expressed miRNAs, there were 10 (Han BB vs. Dorset), 6 (Han BB vs. Han++) and 16 (Han++ vs. Dorset) known miRNAs in sheep species. To predict novel miRNA candidates regulating mRNA, RNAfold was applied to predict the secondary structure of the inverted repeat^20^. Target genes of the differentially expressed miRNAs were predicted using the miRanda program. A summary of the novel miRNAs is shown in Table 2.

### GO and pathway enrichment analysis

A GO enrichment analysis of the DEGs and target genes of the miRNAs was performed to identify GOs with higher confidence. We found that most of the enriched GOs were associated with up-regulated genes in Han BB vs. Dorset, namely neutrophil chemotaxis and calcium ion binding (Figure S1). In addition, the most enriched GO targeted by the down-regulated transcripts in Han BB vs. Dorset was multicellular organismal development.

Pathway enrichment analysis indicated that different pathways were significantly enriched among the up-regulated and down-regulated transcripts among three comparison groups (Figure S2). Additionally, GO and pathway analyses of targets of the differentially expressed miRNAs were also performed (Figures S3 and S4). For example, the targets of miRNAs identified from Han BB compared with Dorset were enriched in categories including memory, protein binding and extracellular regions. Target genes identified from Han++ were associated with the prostaglandin biosynthetic process. The enriched GOs and pathways of the target genes were different from those of the DEGs.

### Path-act-network analysis

To further identify the influence of differentially expressed mRNAs on fecundity, we performed an analysis of DEGs based on the KEGG database and built a pathway interaction network (Figure S5). Through analysing the interactions among the significant pathways, we found that metabolism was the most important pathway in Han BB compared with Dorset sheep. However, the interaction networks of DEGs identified from two other groups were obviously simple.

### Gene-act-network analysis

**Figure 1:**
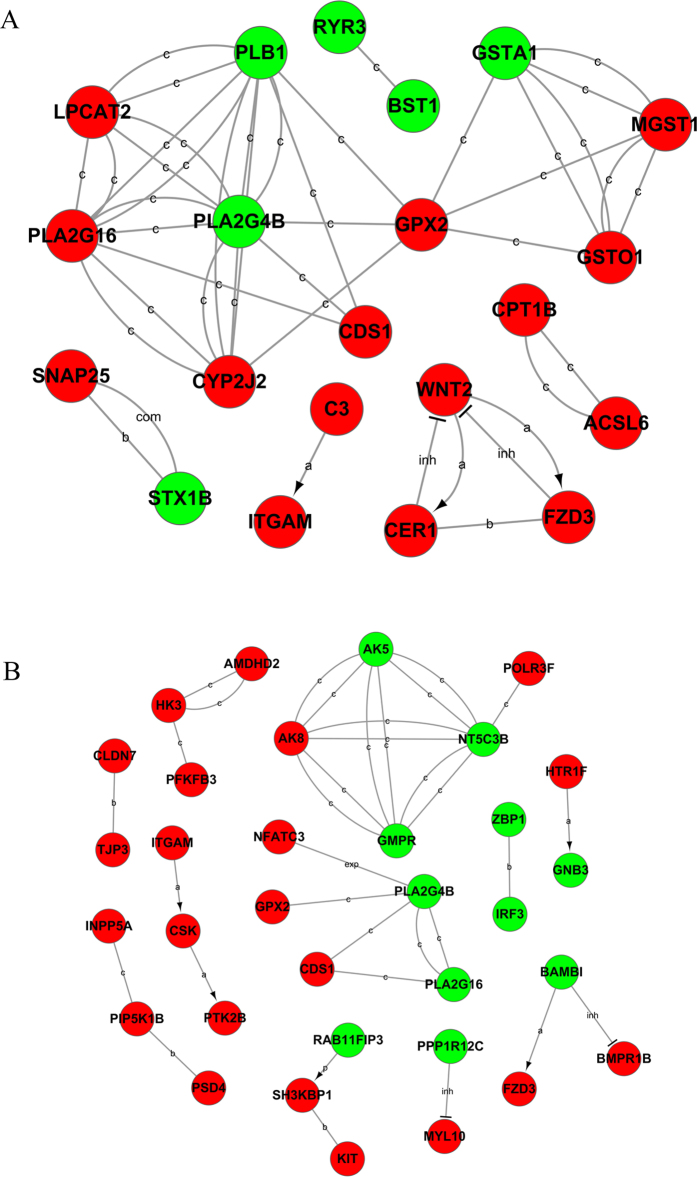
Gene-act network of differentially expressed genes according to pathways in the database. Red circles represent up-regulated genes, and green circles represent down-regulated genes. (**A**) Han BB vs. Dorset; (**B**) Han++ vs. Dorset.

It is valuable to explore the relationship among DEGs after functional analysis. We built a gene-act-network based on the relationships among them according to the STRING database (Fig. 1). Both up- (red circle) and down-regulated (green circle) genes were included in these networks. The results indicated that Phospholipase A2, Group IVB (*PLA2G4B*) was at the core of the interaction network in both Han BB vs. Dorset (Fig. 1A) and Han++ vs. Dorset (Fig. 1B) groups.

### Construction of the miRNA-lncRNA-mRNA networks

**Figure 2:**
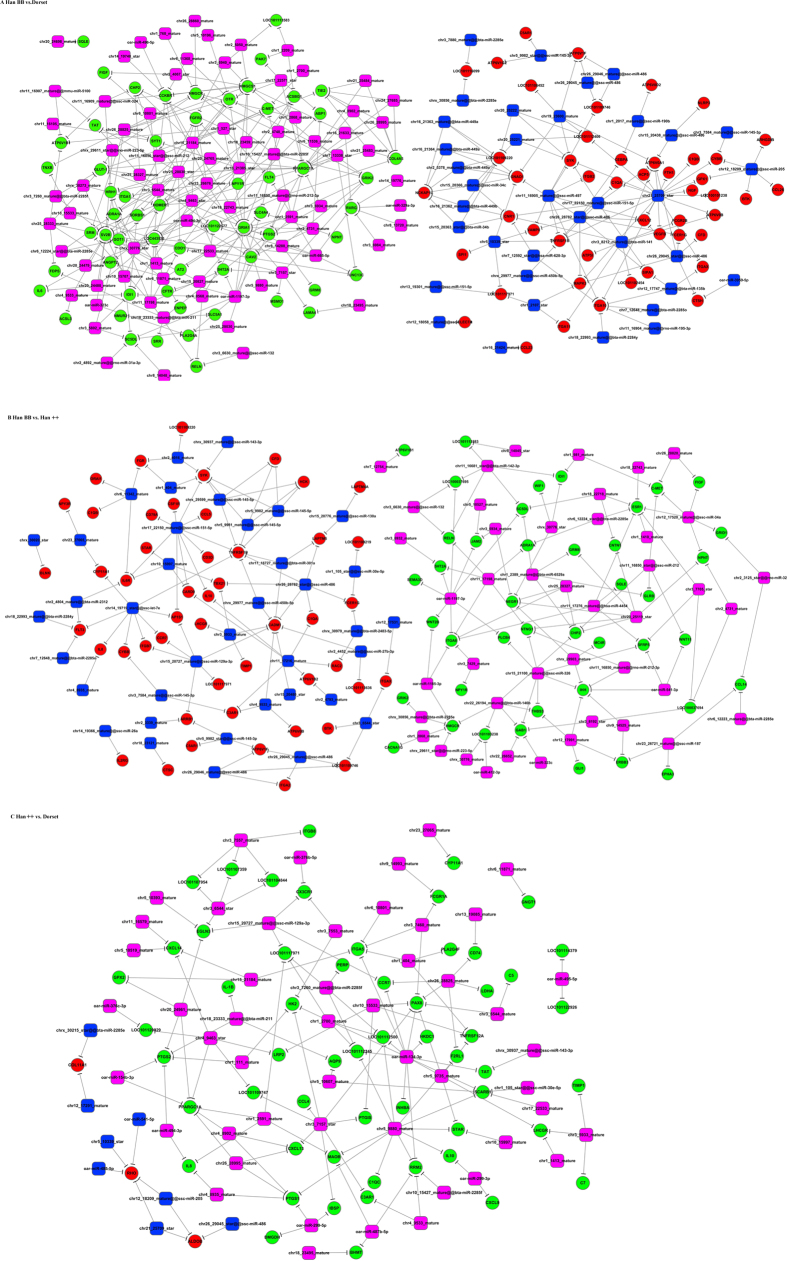
Co-expression networks of differentially expressed miRNAs and mRNAs from each comparison group. Green circles represent down-regulated mRNAs, and red circles represent up-regulated mRNAs. Blue diamonds indicate down-regulated miRNAs and purple diamonds indicate up-regulated miRNAs. (**A**) Han BB vs. Dorset; (**B**) Han BB vs. Han++; (**C**) Han++ vs. Dorset.

To better illustrate these relationships between novel miRNAs and mRNAs, functional networks of miRNA-mRNA pairs were constructed (Fig. 2). If a candidate miRNA and its putative target showed a negative correlation, the gene was identified as a likely true target of the analysed miRNA. In co-expression networks, most miRNAs target several mRNAs, but some miRNAs target only one mRNA. For example, up-regulated oar-miR-1197-3p targeted nine genes, but down-regulated oar-miR-3959-5p targeted only one gene. Moreover, down-regulated Glutamate Receptor, Ionotropic, AMPA1 (*GRIA1*) was regulated by 14 differentially expressed miRNAs including oar-miR-665-5p and oar-miR-1197-3p.

**Figure 3:**
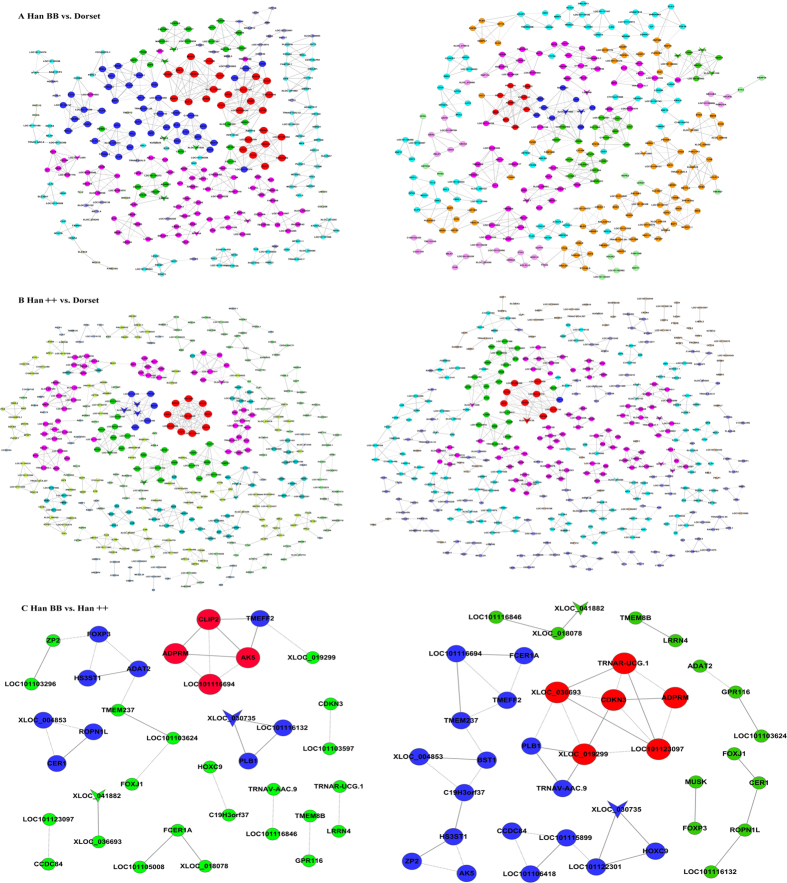
Co-expression networks of fecundity-associated genes and co-regulated lncRNAs. Inverted triangles denote lncRNAs and circles denote mRNAs. Nodes with the same colour represent genes with similar co-expression ability. Node size represents the node degree. (**A**) Han BB vs. Dorset; (**B**) Han BB vs. Han++; (**C**) Han++ vs. Dorset. Han BB, Small Tail Han ewes with genotype BB (HanBB) and genotype++ (Han++); Dorset, Dorset sheep.

lncRNA-mRNA networks in each of two separate groups were constructed based on the correlation analysis with the DEGs and differentially expressed lncRNAs (Fig. 3). LncRNA-mRNA pairs with Pearson correlation coefficients not less than 0.99 were selected to construct the network using Cytoscape. Meanwhile, positive and negative pairs were calculated in each comparison group. The overall structures of the networks of samples and controls were not significantly different in the number of nodes and connections but were significantly different in the degree of nodes (Table 3).

**Figure 4:**
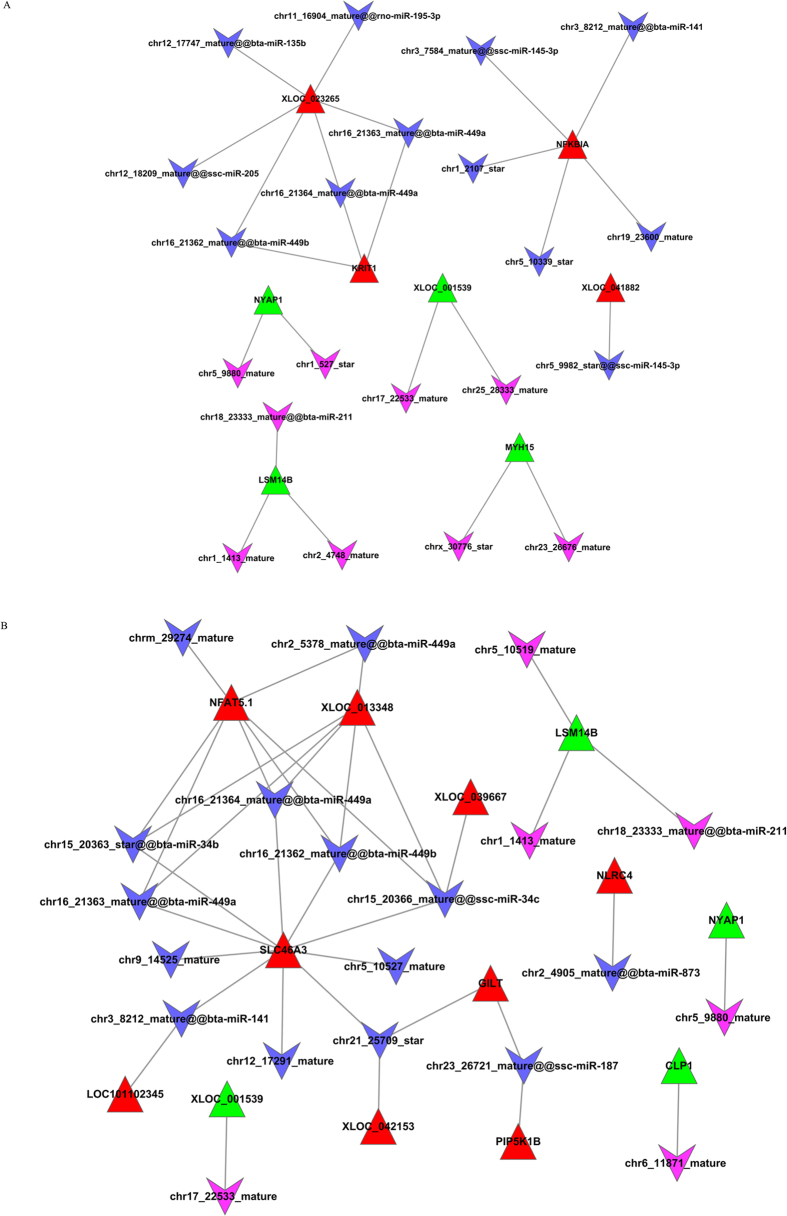
The interaction network between lncRNAs and miRNAs. The red triangle nodes represent the up-regulated lncRNAs, and green represents down-regulated lncRNAs. The purple VEE node represents up-regulated miRNA, and blue represents down-regulated miRNA. (**A**) Han BB vs. Dorset; (**B**) Han++ vs. Dorset.

**Figure 5:**
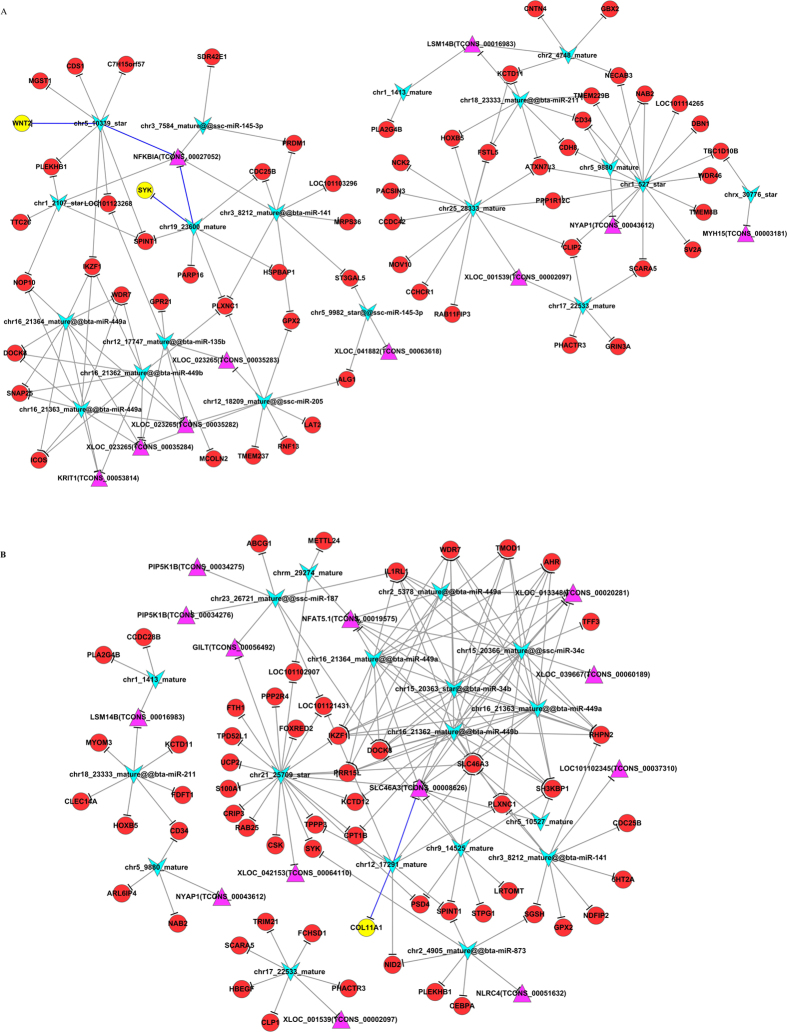
miRNA-lncRNA-mRNA interaction networks. The triangle nodes represent lncRNAs, the circle nodes represent mRNAs, and the VEE nodes represent miRNAs. The yellow nodes represent important mRNAs. (**A**) Han BB vs. Dorset; (**B**) Hans++ vs. Dorset.

To construct the miRNA-lncRNA regulating networks, we predicted the miRNA binding sites on these identified differentially expressed lncRNAs using the RegRNA 2.0 software. Both known and newly predicted miRNAs were analysed, but there were few negative pairs between known miRNA and lncRNAs. We identified 24 and 36 miRNA-lncRNA pairs in the Han BB vs. Dorset and Han++ vs. Dorset groups, respectively (Fig. 4). However, no negative pair was identified in Han BB vs. Han++. Finally, miRNA-lncRNA-mRNA interaction networks were constructed (Fig. 5). The network of Han BB vs. Dorset was composed of 89 nodes and 132 edges, and the nodes included 19 miRNAs, 10 lncRNAs and 60 mRNAs (Fig. 5A). There were 89 nodes and 166 edges in the network of Han++ vs. Dorset, consisting of 18 miRNAs, 13 lncRNAs and 58 mRNAs (Fig. 5B). Furthermore, we found that the miRNAs could bind to one or more lncRNAs.

### Validation of RNA-Seq data by real-time PCR

To validate the RNA-Seq data, we selected 5 differentially expressed miRNAs and 4 lncRNAs to determine the expression of these RNAs by real-time PCR (Figure S6). The expression of each miRNA or lncRNA in the Han BB and Han++ groups was compared with that in the Dorset group. As a result, these differentially expressed miRNAs and lncRNAs may contribute to the fecundity differences among these sheep groups.

## Discussion

Previously, few reports have described miRNA expression profiles for sheep fecundity, and there are no studies of the association of lncRNA expression with sheep prolificacy. This study examined the expression of miRNAs and lncRNAs in sheep ovaries using next generation sequencing and found that miRNAs and lncRNAs were differentially expressed in each comparison. Further analyses of the interaction networks of miRNAs, lncRNAs and mRNAs indicated that differentially expressed miRNAs may play key roles in sheep fecundity by regulating mRNA and lncRNA expression. For example, oar-miR-665-5p was differentially expressed in Han BB sheep compared with Dorset sheep, which is consistent with the result reached in our previous study^9^. Meanwhile, the expression of *GRIA1* was negatively correlated with that of oar-miR-665-5p. Interestingly, a polymorphism in *GRIA1* has been shown to be related to antral follicle number and fertility in cows^21^. Additionally, oar-miR-411a-5p and oar-miR-1197-3p were also differentially expressed in Han BB sheep, and they were also found to be abnormally expressed in goat ovaries^22^. Importantly, oar-miR-1197-39 was also negatively associated with *GRIA1* in the current study, suggesting its key role in sheep fecundity. Notably, rno-miR-34b-3p has been found to be specifically expressed in rat testes^23^, whereas we found it to be differentially expressed in Han BB sheep compared with Dorset group, suggesting that the miRNA may be related to sheep fecundity. Importantly, the integrative analysis of mRNAs and miRNAs identified several miRNA-mRNA pairs, which may contribute to the understanding of genetic regulation relationships. Compared with Dorset sheep, we found that oar-miR-134-3p was differentially expressed in Han++ sheep. Previously, oar-miR-134-3p was indicated to be dysregulated in sheep skeletal muscle^24^. Interestingly, Paired Box 8 (*PAX8*) was found to be negatively correlated with oar-miR-134-3p but also regulated by several other miRNAs. *PAX8* gene function has been suggested to be needed for follicular cells of the thyroid gland, and PAX8 rearrangement may contribute to thyroid tumours^25^,^26^. Combining the aforementioned evidence, differentially expressed miRNAs may play an important role in sheep breeding.

More importantly, we analysed three sheep transcriptomes and profiled the differentially expressed genes according to their GO terms, pathway-act network, gene-act-network and co-expression network. The combination analysis of the lncRNAs and mRNAs suggested similar patterns in the samples compared with the control group but a dissimilar degree of nodes. These results suggest that differentially expressed lncRNAs contribute to sheep prolificacy by regulating the expression of different genes. Although the detailed functions of these genes involved in sheep prolificacy remain largely unknown, the differentially expressed lncRNAs may help us illuminate their roles in co-expression networks. Our co-expression network analysis indicated a similar regulatory relationship in Han BB compared with Han++. There were only two lncRNAs in the networks, including XLOC_030735 and XLOC_041882, which are connected to two and one differentially expressed mRNAs, respectively. In addition, three differentially expressed lncRNAs in Han++ compared with Dorset sheep, including XLOC_029260, the NLR Family, CARD domain containing 4(*NLRC4*) and oxidized low density lipoprotein (lectin-like) receptor 1(*OLR1*), were each connected to nine DEGs. These results indicate that these lncRNAs may exert key functions in their corresponding networks and may thus contribute to sheep fecundity. LncRNAs participate in the regulation of gene expression through several pathways, such as targeting transcription factors, directing methylation complexes, initiating chromatin remodelling and blocking nearby transcription^27^. Importantly, lncRNAs have been shown to be involved in plant and animal fecundity^28^,^29^. This study provides new insight into the lncRNAs involved in sheep prolificacy.

To explore the function of lncRNAs acting as miRNA targets, miRNA-lncRNA-mRNA interaction networks were constructed. We found that these molecules formed complex regulatory networks and participated in many biological processes. Moreover, with the presentation of the hypothesis of competing endogenous RNA (ceRNA), our findings suggest that lncRNAs could be regulated by miRNAs and thereby favour the expression of repressed mRNA targets. For example, chrx_30776_star and myosin heavy chain 15 (MYH15) interact with each other, indicating that MYH15 may combine with chrx_30776_star through competition with other target mRNAs.

Furthermore, GO and pathway analyses were conducted to identify biological functions enriched among the target mRNAs of differentially expressed miRNAs. We found that target genes of the miRNAs were mainly involved in metabolic pathways, carbon metabolism and RNA transport. Notably, metabolic pathway was associated with 706 differentially expressed transcripts, which suggests that sheep fecundity is mainly associated with metabolic processes. Interestingly, metabolism is enriched in mammalian development including prolificacy.

## Conclusions

In the present study, we constructed interaction networks among many miRNAs, lncRNAs and mRNAs for the identification of lncRNAs and miRNAs associated with sheep fecundity. Our study lays a solid foundation for elucidating the regulatory mechanisms of lncRNAs and miRNAs in sheep and provides a unique source for exploring lncRNAs as miRNA targets in the future. These deregulated miRNAs and lncRNAs may play a critical role in sheep prolificacy. Taken as a whole, our findings demonstrate that the transcriptomic study of sheep ovaries generates novel insights into sheep fecundity, resulting in the implication of many previously uncharacterized elements in developmental biology.

## Materials and Methods

### Ethics statement

All of the procedures involving animals were approved by the Animal Care and Use Committee at the Institute of Animal Sciences, Chinese Academy of Agricultural Sciences, where the experiment was conducted. All of the experiments were performed in accordance with the relevant guidelines and regulations set by the Ministry of Agriculture of the People’s Republic of China.

### Sample preparation

Small Tail Han sheep (Han) and Dorset sheep (Dorset) were raised in Qingdao Aote Sheep Farm (Shandong, China) under similar conditions of free access to food and water in natural lighting. Two types of Han sheep were used, Han BB and Han++, which were considered as two groups of high-fecundity Han sheep. Dorset sheep were used as the control low-fecundity group. All samples were obtained according to the methods in a previous study^9^. All ovary samples were stored at −70 °C for total RNA extraction.

### Construction of sequencing libraries and RNA-Seq

Total RNA was extracted from the ovaries of Han sheep and Dorset sheep using Trizol reagent (Invitrogen, Carlsbad, CA). The preparation of the whole transcriptome library, small RNA library and deep sequencing were conducted by Novel Bioinformatics Co., Ltd (Shanghai, China). The transcriptome library was constructed using Illumina TruSeq Stranded Total RNA with Ribo-Zero Globin (Illumina, San Diego, CA, USA) according to the manufacturer’s instructions. The small RNA libraries were constructed according to the Illumina TruSeq^TM^ Small RNA Sample Preparation protocol. Library size and purity were measured using an Agilent 2100 system (Agilent, California).

### Read mapping and annotation

RNA-Seq reads from each sample were aligned to the oar 3.1 sheep reference genome with TopHat using the default settings^30^. Only uniquely mapped reads were used for gene expression analysis. According to the rigorous significance test for the digital gene expression profiling described previously, the DESeq package was used to identify significantly differentially expressed genes (DEGs)^31^. False discovery rate (FDR) was used for the error rate adjustment in multiple significance tests^32^. If the fold change was >1.5 or <0.667, and the FDR was <0.05, the miRNAs and lncRNAs were considered to be differentially expressed.

### Target gene prediction for differentially expressed miRNAs

To remove the adaptor sequences and the low-quality sequences, the raw small RNA reads were processed with the fastx toolkit. After alignment of small RNA reads with the Sanger miRBase, piRNA, ncRNA and Rfam databases, the differentially expressed miRNAs were screened out when |log_2_(fold change)| >1 and *P* < 0.05. To predict novel miRNAs, the unannotated reads were analysed with the miRCat program^33^. The miRanda program was employed to predict potential target genes of the differentially expressed miRNAs^34^. Additionally, a correlation analysis between miRNAs and mRNAs was performed. If the candidate miRNAs and the putative targets showed a negative correlation, the gene was identified as likely a true target of the analysed miRNA. To better illustrate these relationships between novel miRNAs and mRNAs, functional networks of miRNA-mRNA pairs were constructed.

### Functional enrichment analysis

The GO project provides a controlled vocabulary to describe genes and gene products covering three domains: molecular function, cellular component and biological process. To understand the potential roles of DEGs and miRNA target genes, GO categories were applied in this study^35^. The KEGG database was used to identify significant pathways for predicted target genes^36^. *P* < 0.05 was used as the threshold.

### Pathway-act-network analysis

Path-act-network analysis was performed to identify the interactive network among the pathways enriched for differentially expressed mRNAs based on the KEGG database, including membrane transport, metabolism, cell cycle and signal transduction pathways. The genes we selected may be involved in two or more signalling pathways. The pathways were presented with Cytoscape^37^.

### Gene-act-network analysis

The STRING database was employed to conduct gene-act-network analysis to reveal the network of the differentially expressed mRNAs based on relationships extracted from the database, including expression (exp), activation (a), binding (b), inhibition (inh), catalysis (c) and compound (com)^38^.

### Construction of miRNA-lncRNA-mRNA networks

To infer the function of lncRNAs in sheep fecundity, networks were constructed based on complementary pairs between miRNA and mRNAs and between miRNAs and lncRNAs. The nodes in the networks included miRNAs, lncRNAs and mRNAs. The miRNA-lncRNA-mRNA networks were visualized with Cytoscape 2.8^37^.

### Quantitative PCR

Following total RNA extraction, ovary samples were reversely transcribed with appropriate primers to generate cDNA. Real-time PCR was performed in triplicate with reaction volume of 10 μl (5 μl of 2 × LightCycler 480 SYBR Green I Master Mix, 0.2 μl of PCR forward primer, 0.2 μl of PCR reverse primer, 1 μl of cDNA and 3.6 μl of nuclease-free H_2_O) and incubated (95 °C for 10 min, then 40 cycles at 95 °C for 10 sec, 60 °C for 30 sec). After PCR amplification, a melt curve analysis was carried out to confirm the reaction specificity, with 18S rRNA (for lncRNA) and 5S (for miRNA) used as internal controls.

### Statistical analyses

All of the data are presented as the mean ± SD. When comparisons were made, Student’s t-test was performed, and p < 0.05 was considered statistically significant.

## Additional Information

**How to cite this article**: Miao, X. *et al*. Ovarian transcriptomic study reveals the differential regulation of miRNAs and lncRNAs related to fecundity in different sheep. *Sci. Rep.* **6**, 35299; doi: 10.1038/srep35299 (2016).

## Supplementary Material

Supplementary Information

## Figures and Tables

**Table 1:** Summary of reads mapping to the sheep transcriptomes.

	Han BB	Han++	Dorset
Number of all reads	46,495,842	56,383,118	50,333,796
Number of bases (in Gb)	5.17	6.05	5.43
Number of mapped reads	36,320,142	49,791,023	44,572,071
% mapped reads	78.1	88.3	88.6
Number of uniquely mapped reads	33,597,314	47,963,451	42,998,557
% uniquely mapped reads	72,3	85.1	85.4

Han BB, Small Tail Han ewes with genotype BB (HanBB) and genotype++ (Han++); Dorset, Dorset sheep.

**Table 2:** Number of differentially expressed coding and non-coding genes identified from each comparison group.

Number of differentially expressed genes	Han BB vs. Dorset	Han++ vs. Dorset	Han BB vs. Han++
mRNAs	2511	1280	2241
lncRNAs	138	138	106
Novel miRNAs	132	98	100

Han BB, Small Tail Han ewes with genotype BB (HanBB) and genotype++ (Han++); Dorset, Dorset sheep.

**Table 3:** Summary of the co-expression networks for the differentially expressed lncRNAs and mRNAs identified from each comparison group.

	Han BB vs. Dorset		Han++ vs. Dorset		Han BB vs. Han++	
	Han BB	Dorset	Han++	Dorset	Han BB	Han++
Number of nodes	282	283	381	397	37	38
Number of positive pairs	422	386	437	357	15	25
Number of negative pairs	310	341	351	356	17	20

Han BB, Small Tail Han ewes with genotype BB (HanBB) and genotype++ (Han++); Dorset, Dorset sheep.
