# Supplementary material for: Ovarian transcriptomic study reveals the differential regulation of miRNAs and lncRNAs related to fecundity in different sheep
Source: Sci Rep. 2016 Oct 12;6:35299. doi: 10.1038/srep35299 (PMC5059661; doi:10.1038/srep35299)
Supplement: Supplementary Information [file srep35299-s1.pdf]

Supplementary information

Ovarian transcriptomic study reveals the differential regulations of miRNAs and lncRNAs related to fecundity in different sheep

Xiangyang Miao* Qingmiao Luo Huijing Zhao Xiaoyu Qin

Institute of Animal Sciences, Chinese Academy of Agricultural Sciences, Beijing, 100193, China

*Corresponding author: Xiangyang Miao Institute of Animal Sciences, Chinese Academy of Agricultural Sciences, Beijing, 100193, China, Tel: 86-10-62895663, Fax: 86-10-62895663 (China), E-mail: miaoxy32@163.com, mxy32@sohu.com

Figure S1 Functional classification of differentially expressed genes identified from each comparison group based on Gene Ontology (GO). GO analysis was conducted to determine the enrichment of genes in biological process (BP), cellular components (CC) and molecular function (MF) with $P< 0.05$. (A) Han BB vs. Dorset; (B) Han BB vs. Han ++; (C) Han ++ vs. Dorset.

Figure S2 KEGG pathway enrichment analysis of differentially expressed mRNAs identified from each comparison group with significance $P < 0.05$. (A) Han BB vs. Dorset; (B) Han BB vs. Han ++; (C) Han ++ vs. Dorset.

Figure S3 Functional classification of target genes of differentially expressed

miRNAs identified from each comparison group based on Gene Ontology (GO). GO analysis was conducted to determine the enrichment of genes in biological process (BP), cellular components (CC) and molecular function (MF) with $P < 0.05$. (A) Han BB vs. Dorset; (B) Han BB vs. Han ++; (C) Han ++ vs. Dorset.

Figure S4 KEGG pathway enrichment analysis of target genes of differentially expressed miRNAs identified from each comparison group with significance $P < 0.05$. (A) Han BB vs. Dorset; (B) Han BB vs. Han ++; (C) Han ++ vs. Dorset.

Figure S5 Pathway-act-network analysis. A, Han BB vs. Dorset; B, Han ++ vs. Dorset; C, Han BB vs. Han ++.

Figure S6 Validation of RNA-Seq data by quantitative real-time PCR. The relative expression level of each lncRNA was normalized to 18S rRNA, and each miRNA was normalized to 5S rRNA.

A Han BB vs. Dorset

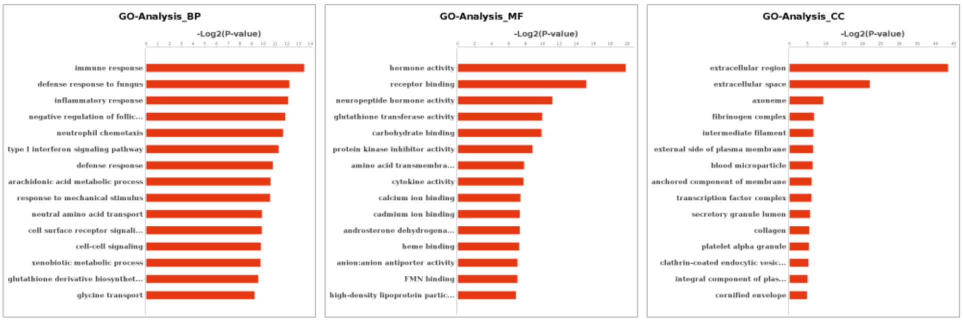

B Han BB vs. Han ++

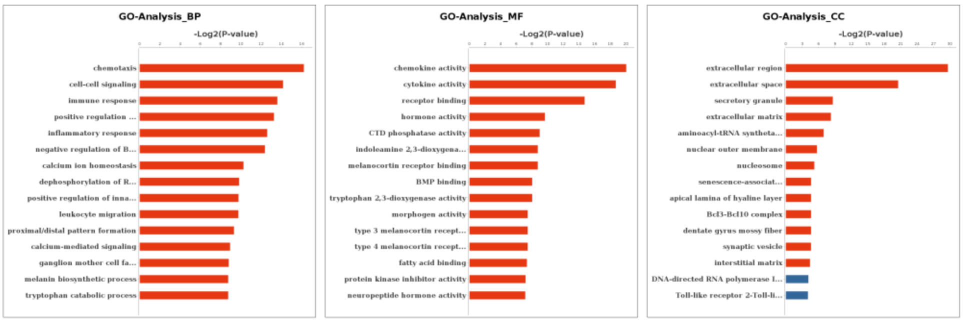

C Han++ vs. Dorset

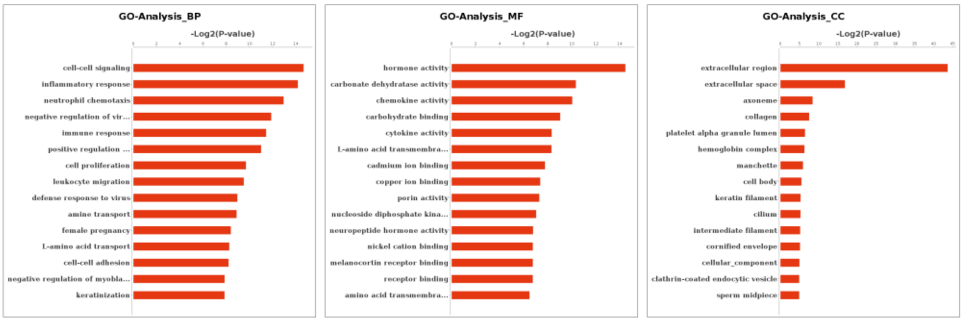

A Han BB vs. Dorset

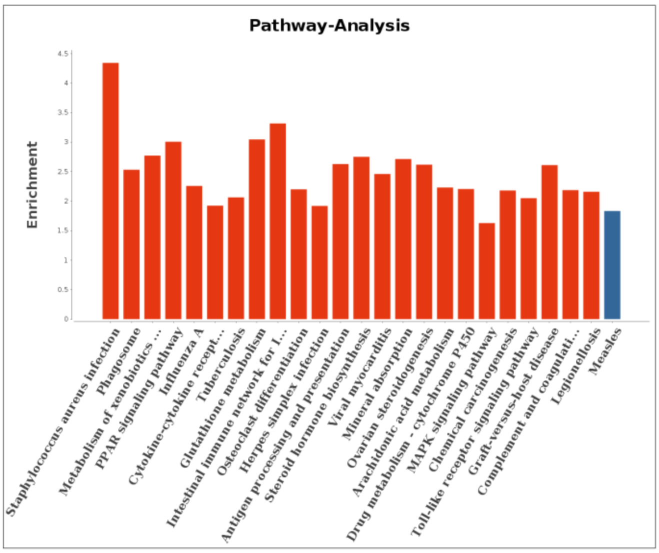

B Han BB vs. Han++

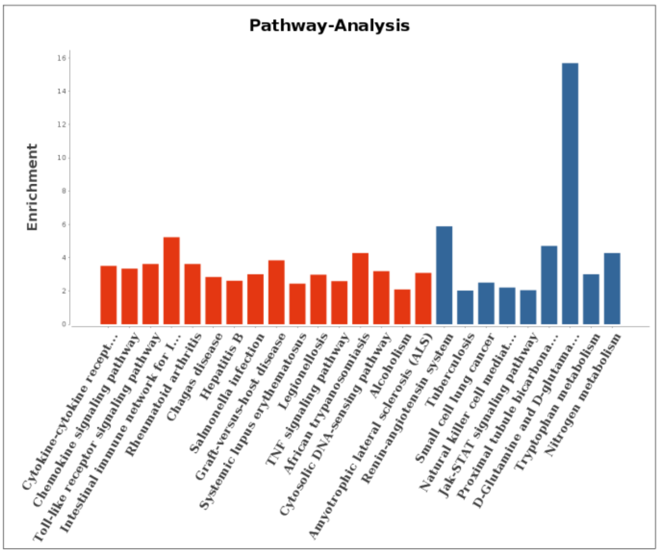

C Han++ vs. Dorset

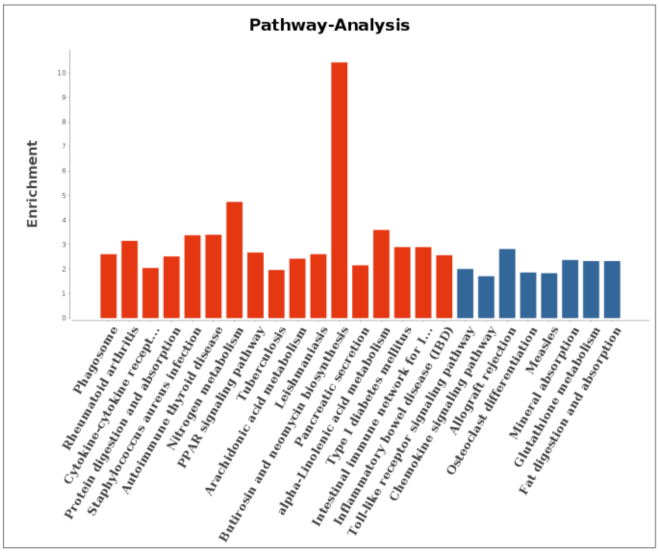

A Han BB vs. Dorset

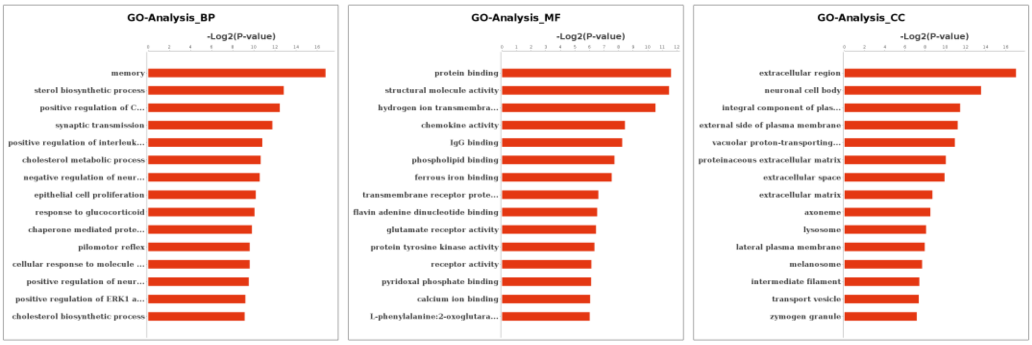

B Han BB vs. Han++

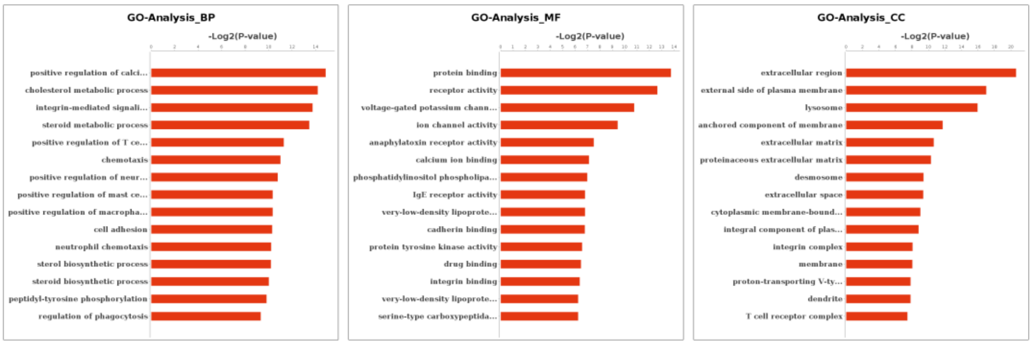

C Han++ vs. Dorset

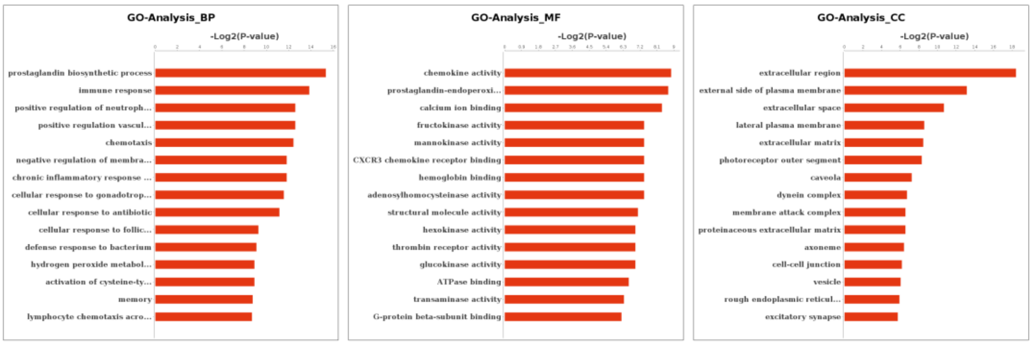

A Han BB vs. Dorset

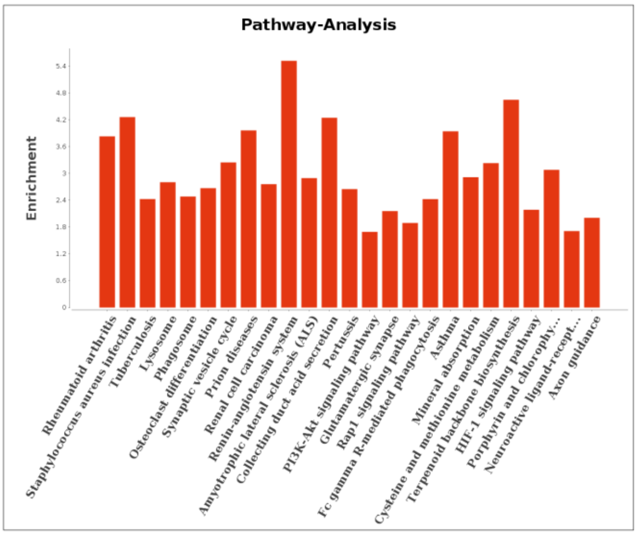

B Han BB vs. Han++

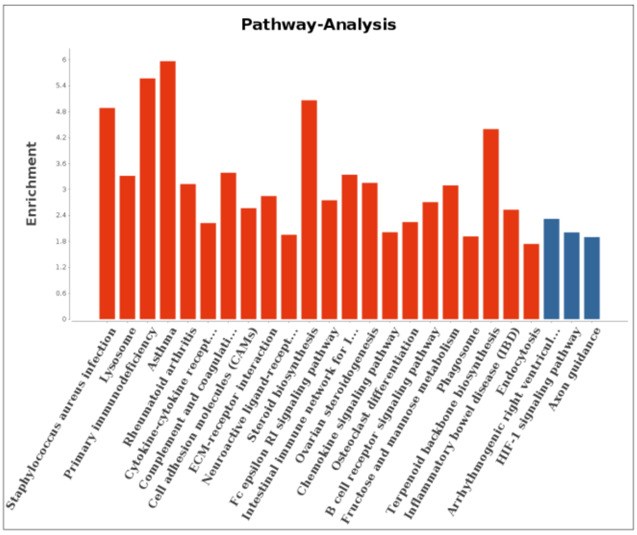

C Han++ vs. Dorset

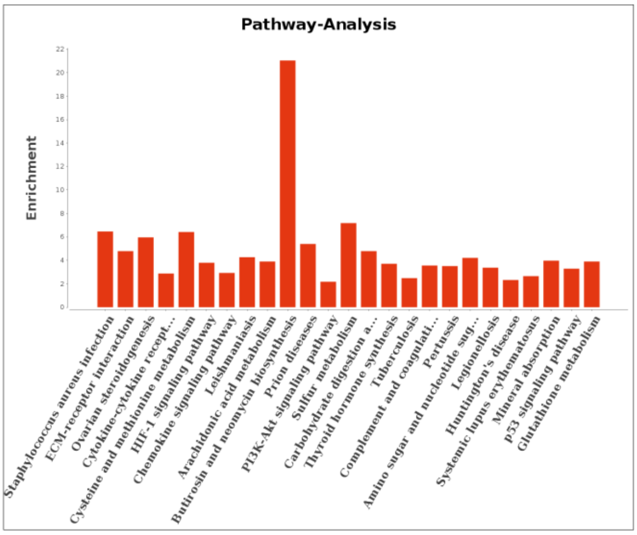

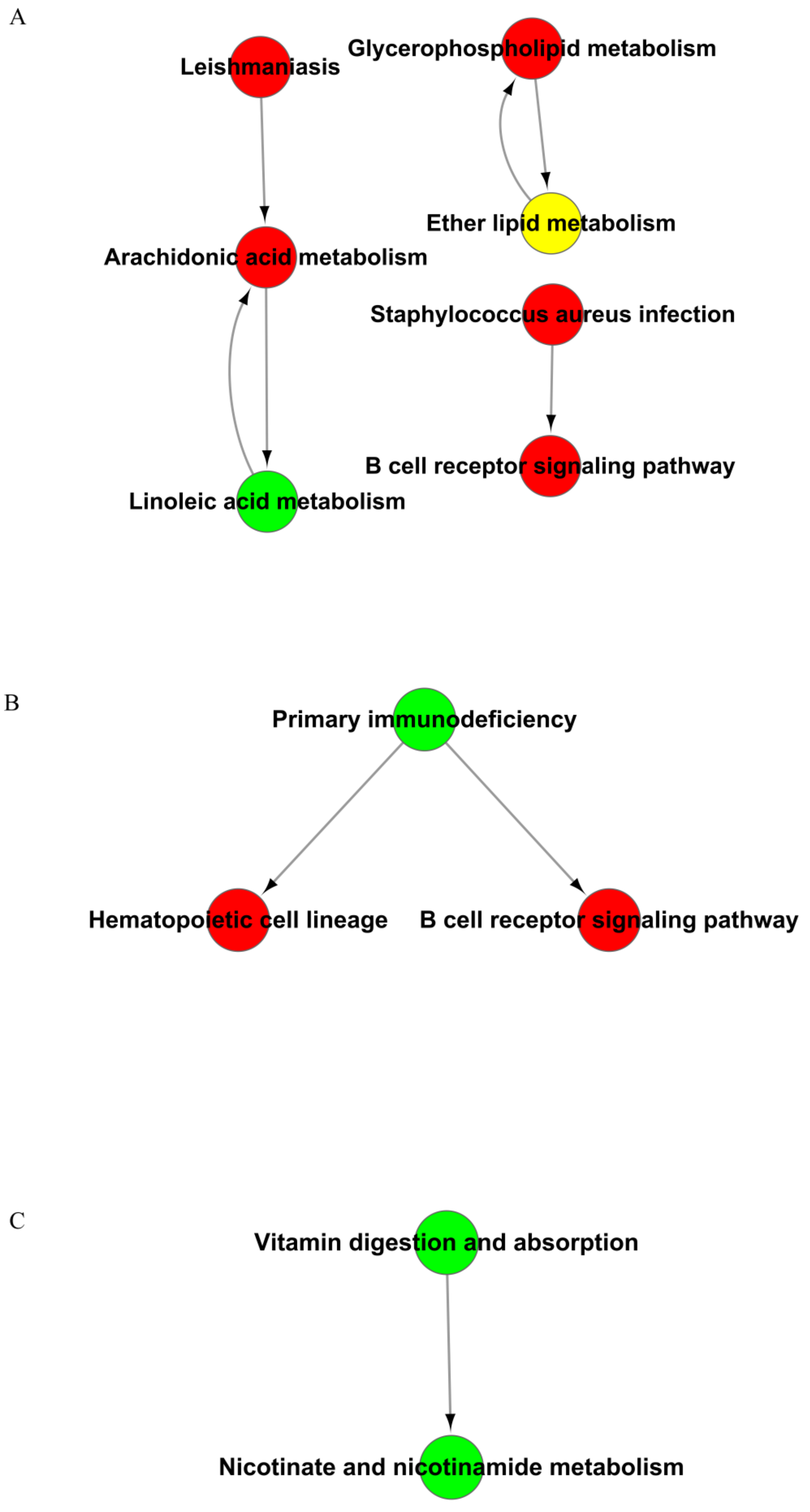
A
Leishmaniasis
Glycerophospholipid metabolism
Ether lipid metabolism
Arachidonic acid metabolism
Staphylococcus aureus infection
B cell receptor signaling pathway
Linoleic acid metabolism
B
Primary immunodeficiency
Hematopoietic cell lineage
B cell receptor signaling pathway
C
Vitamin digestion and absorption
Nicotinate and nicotinamide metabolism

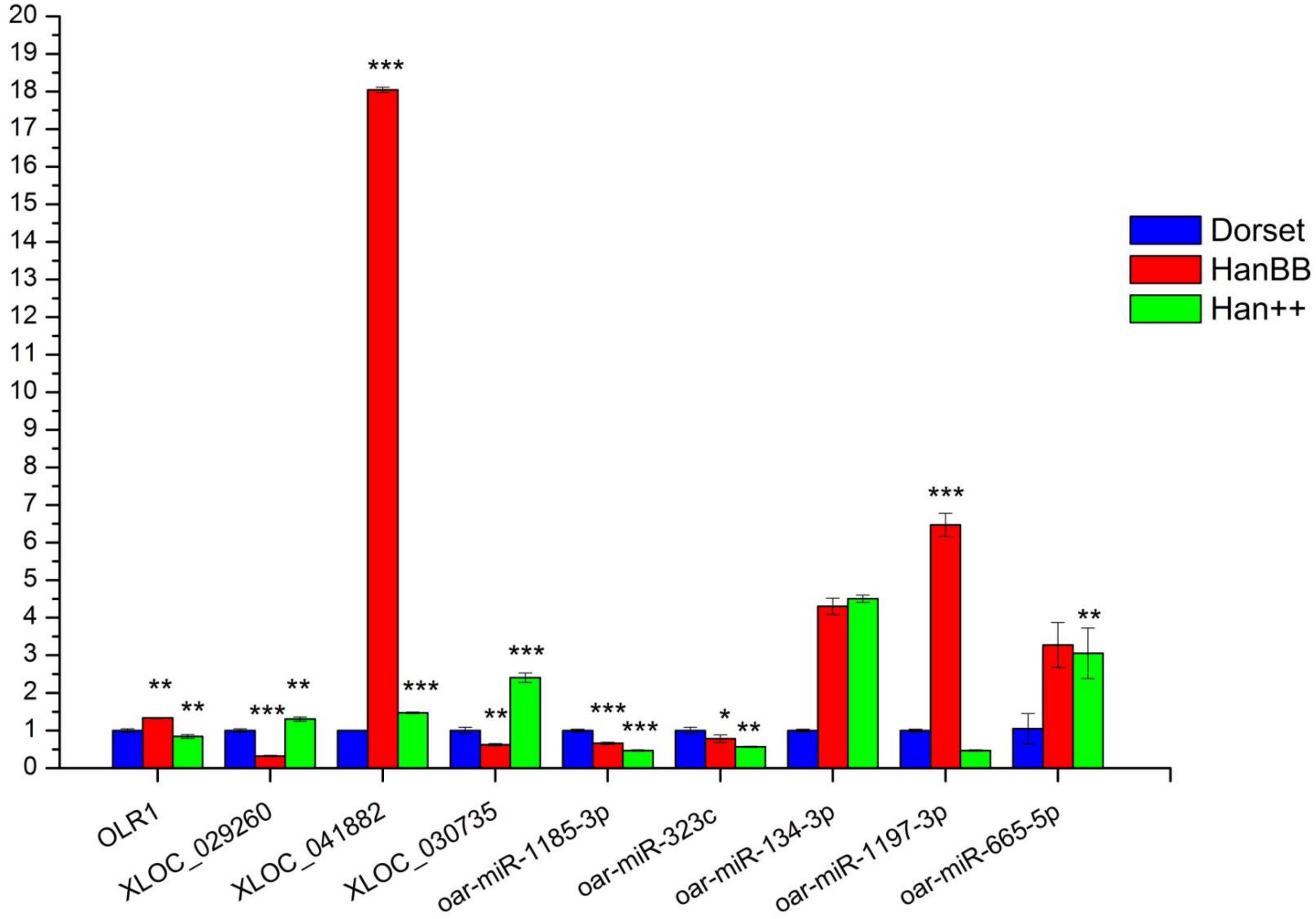
20
19
18
17
16
15
14
13
12
11
10
9
8
7
6
5
4
3
2
1
0
Dorset
HanBB
Han++
***
***
**
**
**
***
***
***
**
***
***
*
**
***
**
OLR1
XLOC_029260
XLOC_041882
XLOC_030735
oar-miR-1185-3p
oar-miR-323c
oar-miR-134-3p
oar-miR-1197-3p
oar-miR-665-5p
